# Computational Model for Therapy Optimization of Wearable Cardioverter Defibrillator: Shockable Rhythm Detection and Optimal Electrotherapy

**DOI:** 10.3389/fphys.2021.787180

**Published:** 2021-12-10

**Authors:** Oishee Mazumder, Rohan Banerjee, Dibyendu Roy, Ayan Mukherjee, Avik Ghose, Sundeep Khandelwal, Aniruddha Sinha

**Affiliations:** TCS Research, Tata Consultancy Services, Kolkata, India

**Keywords:** sudden cardiac death, defibrillation threshold, hemodynamics, myocardial damage, biophysical simulation, deep learning

## Abstract

Wearable cardioverter defibrillator (WCD) is a life saving, wearable, noninvasive therapeutic device that prevents fatal ventricular arrhythmic propagation that leads to sudden cardiac death (SCD). WCD are frequently prescribed to patients deemed to be at high arrhythmic risk but the underlying pathology is potentially reversible or to those who are awaiting an implantable cardioverter-defibrillator. WCD is programmed to detect appropriate arrhythmic events and generate high energy shock capable of depolarizing the myocardium and thus re-initiating the sinus rhythm. WCD guidelines dictate very high reliability and accuracy to deliver timely and optimal therapy. Computational model-based process validation can verify device performance and benchmark the device setting to suit personalized requirements. In this article, we present a computational pipeline for WCD validation, both in terms of shock classification and shock optimization. For classification, we propose a convolutional neural network-“Long Short Term Memory network (LSTM) full form” (Convolutional neural network- Long short term memory network (CNN-LSTM)) based deep neural architecture for classifying shockable rhythms like Ventricular Fibrillation (VF), Ventricular Tachycardia (VT) vs. other kinds of non-shockable rhythms. The proposed architecture has been evaluated on two open access ECG databases and the classification accuracy achieved is in adherence to American Heart Association standards for WCD. The computational model developed to study optimal electrotherapy response is an *in-silico* cardiac model integrating cardiac hemodynamics functionality and a 3D volume conductor model encompassing biophysical simulation to compute the effect of shock voltage on myocardial potential distribution. Defibrillation efficacy is simulated for different shocking electrode configurations to assess the best defibrillator outcome with minimal myocardial damage. While the biophysical simulation provides the field distribution through Finite Element Modeling during defibrillation, the hemodynamic module captures the changes in left ventricle functionality during an arrhythmic event. The developed computational model, apart from acting as a device validation test-bed, can also be used for the design and development of personalized WCD vests depending on subject-specific anatomy and pathology.

## 1. Introduction

Sudden cardiac death (SCD) is a sudden and unpredictable event caused due to loss of cardiac functionality. SCD accounts for the largest cause of natural death in the adult population, causing around 13% of deaths in the overall population and about 36% of deaths in heart failure patients (Smith and Cain., 2006). The leading cause of SCD is primarily attributed to electrical abnormality like ventricular arrhythmia (VA) and ventricular fibrillation (VF) followed by structural cardiac disorders like ischemia. VF is usually lethal within minutes of its inception and if not immediately treated, leads to cardiac arrest (Barraud et al., 2017). Electrical defibrillation is the only effective therapy for such conditions. Electrical defibrillation through wearable cardioverter-defibrillator (WCD) provides a non-invasive therapeutic option for patients during a period when the risk of SCD is changing or unclear (Poole et al., 2008; Sharma et al., 2017).

Wearable cardioverter-defibrillator is mostly recommended to patients who are newly diagnosed with non-ischemic cardiomyopathy with severely reduced left ventricle ejection fraction (LVEF), patients awaiting heart transplantation or in patients with ventricular assist devices, temporary inability to implant an intra-cardiac defibrillator (ICD) or in ambulatory event monitoring, often performed for several weeks in an effort to determine an arrhythmic etiology for syncope (Wan et al., 2013). Similar to ICD, WCD detects ventricular arrhythmic events and delivers a defibrillation shock to terminate VF or tachycardia (>180 bpm) by resetting myocardial potential distribution. Instead of the intracardiac electrogram (EGM) signal, the ECG signal recorded from the body surface is used to detect arrhythmic events. Therapeutic devices like WCD though indispensable, have to maintain very high reliability and accuracies in order to deliver timely and optimal therapy (Epstein et al., 2013). WCD devices are programmed to be autonomous, thus further burdening device complexity. Malfunction in any form while in detection or during shock generation can cause serious injury, which can even be fatal. An effective way of device reliability and performance validation is through computational model aided trials (Ariful et al., 2016). For WCD, computer-aided validation processes could evaluate device performance in virtual trials and benchmark the device settings to suit personalized requirements. In this regard, two separate aspects require validation and bench-marking: the classification accuracy of detecting shockable rhythm from non-shockable rhythm and shock voltage profile optimization based on a personalized requirement to optimize shock efficacy.

The central requirement of autonomous WCD devices is the detection of VF by means of reliable detection algorithms. Over the past decades, special focus has been given toward developing efficient algorithms that can correctly detect VF abnormality, especially in real-time (Ayala et al., 2014; Figuera et al., 2016). Different large-scale machine learning (ML) methods have been explored for ECG beat classification identifying shockable rhythm (Jekova, 2000; Amann et al., 2005). In spite of the high accuracy of classification, incorporating complex feature measurement within the WCD setting is a challenge. WCD shock detection algorithm requires real-time analysis with minimal decision delay, low complexity, and low memory requirement for computations that presents a certain risk of poor feature quality due to inaccurate delineation of ECG waves, filtering, or approximations (Aramendi et al., 2010). As an alternate, several self-learning approaches based on the deep neural network have been proposed recently (Zhong et al., 2020) and are now widely applied on arrhythmia classification using Convolutional Neural Network (CNN) (Lee et al., 2019). The CNN-based arrhythmia classification could eliminate the cumbersome requirement of criteria selections and parameters setting in traditional ML-based arrhythmia detection methods while achieving high detection accuracy. Some notable prior arts (Silva et al., 2019) implementing CNN architecture for arrhythmia classification, reports the use of various architectural layers (Kwon et al., 2018), attention on noise removal, use of LSTM networks (Krasteva et al., 2020), etc. The most recent work reporting the highest accuracy to date uses a bidirectional LSTM (bi-LSTM) instead of unidirectional LSTM (Jeon et al., 2020).

Irrespective of high detection accuracy and type of defibrillator, strong shocks that are required during defibrillation are reported to have serious adverse effects, most prominently *via* electroporation that may initiate post-shock arrhythmia (Colley et al., 2019). Strong shocks can also potentially cause myocardial damage, giving rise to mechanical dysfunction (stunning), increase in contractility, and development of hemodynamically mediated symptoms (Qiana et al., 2018). Hence, it is extremely important to tune and optimize the shock energy to get the desired effect. The mechanism of defibrillation has been studied extensively in recent years, mostly for ICD placements (Stinstra et al., 2008; Onofrio et al., 2018). Computational models analyzing defibrillation mechanism and the after-effect of shock voltage in the myocardium can provide an in-depth understanding of the fibrillation mechanism and help in optimizing the defibrillation threshold (Stinstra et al., 2007). The distribution of electric fields in the heart is closely related to defibrillation outcomes. Three dimensional cardiac models like the volume conductor models coupled with Finite Element Modeling are well suited to reflect the electric field distribution in myocardium substrate (Stinstra et al., 2010; Trayanova et al., 2011; Tate et al., 2018).

Prior art lists sufficient methods of classifying shockable and non-shockable rhythms but for defibrillator performance validation, an integrated pipeline that could classify shockable rhythm as well as validate the shockable energy delivery efficacy is the need of the hour. The shock delivery circuit of WCD generates very strong fields of fixed energy or current to stop the arrhythmic propagation by resetting the myocardial potentials to a depolarized state (Morgan et al., 2009). It has been observed that field distribution required to provide defibrillation effect is greatly dependent on subject-specific parameters like torso geometry, trans-thoracic impedance, cardiac structure, etc. (Hatib et al., 2000). A computational pipeline that could integrate the aspects of shock identification and pre-plan personalized shock delivery can be extremely useful as a WCD device validation. Mathematical modeling and computer simulation can efficiently accelerate the process of optimizing and testing of WCDs. A computational model-based validation approach could evaluate device performance on virtual trial and benchmark the device setting to suit personalized requirements.

**Figure 1:**
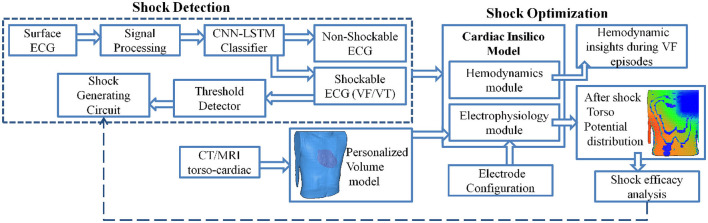
Schematic representation of the computational model to analyze Wearable cardioverter-defibrillator (WCD) efficacy.

In this article, we propose a computational pipeline for WCD validation, both in terms of shock classification and shock optimization. The schematic representation of the proposed computational framework is shown in Figure 1. The computational model is an *in-silico* cardiac model integrating cardiac functionality in terms of hemodynamics and electrophysiology, encompassing biophysical simulation to compute the effect of shock voltage on myocardial potential distribution. We propose a CNN-LSTM architecture for the classification of VF, VT (shockable), and other (non-shockable) rhythms. The proposed network is evaluated on two open-access databases, the CUDB and the VFDB databases. A 3D cardiac computational model in line with the volume conductor model is developed utilizing high definition torso-cardiac MRI. This model is used to study the variation of shock efficacy by varying plausible electrode configurations. A novel metric is designed for quantifying the shock efficacy computed using the energy required to obtain DFT and extent of myocardial damage. Along with the biophysical modeling aspect, the cardiac computational model also integrates the hemodynamics functionality that closely replicates the dynamic changes in left ventricular functions during VF/VT episodes, thus providing key physiological insights. Novelty and uniqueness of the proposed computational pipeline for shock classification and distribution analysis lies in incorporating a CNN-LSTM overlapping window algorithm, deriving defibrillation efficacy metric for optimal electrotherapy, and inclusion of hemodynamic insights during VF initiation and subsequent termination. Such concepts have not been proposed earlier for WCD and have the potential to enhance conventional WCD functioning in terms of device validation and personalization.

## 2. Materials and Methods

The proposed computational framework is divided into three major sections involving the key features of the proposed model which are

- CNN-LSTM based shockable rhythm classifier architecture with both non-overlapping and overlapping window variations
- Biophysical modeling of shock propagation and shock efficacy index generation
- Capturing hemodynamics changes during VF/VT episodes and recovery

Subsequent sections concentrate on the development and integration of each of these features in a computational model incorporating the functionality of a standard WCD. The modeled WCD referred to in this paper is the WCD model of Zoll electronics (WCD system, LifeVest, ZOLL, Pittsburgh, PA, USA) (Reek et al., 2017). The basic working of WCD can be found in the Supplementary Material.

### 2.1. Dataset and ECG Pre-processing

The classification algorithm is designed and validated using two publicly available datasets, the MIT-BIH Malignant Ventricular Arrhythmia database (VFDB) (Greenwald, 1986) and the Creighton University Ventricular Tachycardia database (CUDB) (Nolle et al., 1986). The VFDB dataset contains 30-min long Holter ECG record files belonging to 22 subjects. The CUDB dataset contains 8-min long ECG records collected from 35 patients who have experienced sustained episodes of lethal VA. The sampling rate for all recordings is 250*Hz*.

**Figure 2:**
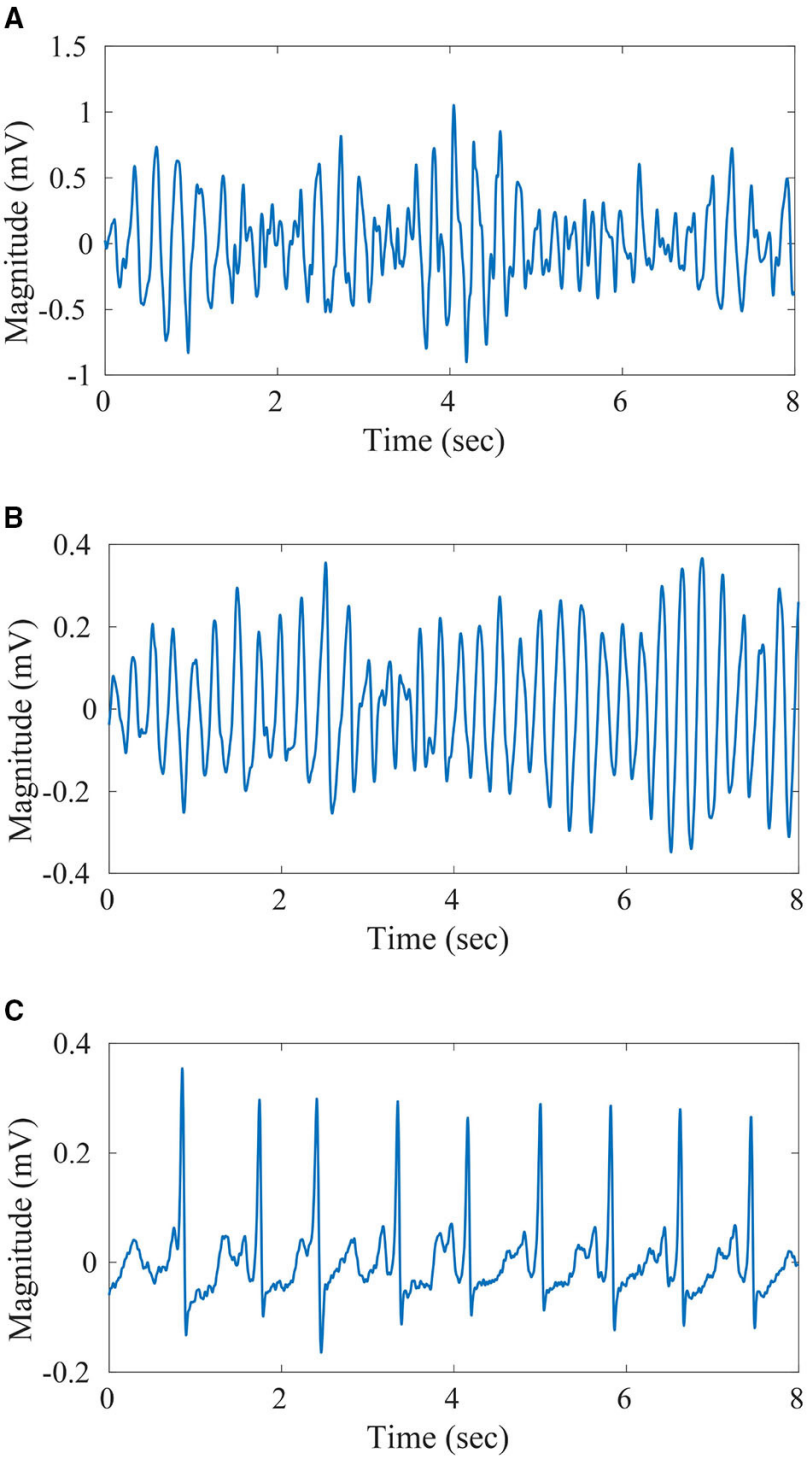
Sample ECG-**(A)** 8 s duration Ventricular Tachycardia (VT) signal, **(B)** 8 s duration VF signal, **(C)** 8 s duration non-shockable signal.

A pre-processed version of the ECG recording datasets, as discussed in Krasteva et al. (2010) and Bisera et al. (2008), have been utilized for the design and validation of the proposed algorithm. The preprocessing steps followed are mean subtraction, moving average filtering [*order* = 5], a high-pass filter with *f*_*c*_ = 1*Hz* to eliminate drift suppression, and low-pass Butterworth filter with *f*_*c*_ = 30*Hz*. Further, noise and artifacts have been excluded from the datasets along with intermediate rhythms like slow VT (<150 bpm) and fine VT. Also, recording segments with minimal electrical activity have also been excluded from the datasets. With all the preceding pre-processing steps applied, the resultant recording is split into windows of different lengths namely, 2, 4, 6, and 8 s. Further, windows with uniform labeling only have been retained for use. The details regarding the final count of windows generated through the process for the different classes under consideration (VT, VF, and Others) are given in Table 1. These instances have been used for training and validation of the proposed algorithm in a k-fold cross-validation framework. Plots of the 8 s recordings with labels VT, VF, and non-shockable rhythm are given in Figures 2A–C, respectively.

**Table 1:** Dataset segmentation details.

	**CUDB**			**VFDB**		
**Segment length** **(s)**	**NSh**	**VF**	**VT**	**NSh**	**VF**	**VT**
2	6,075	120	1,390	16,005	1,473	1,597
4	2,986	53	663	7,861	702	784
6	1,959	31	422	5172	446	516
8	1,446	21	302	3,823	326	377

Apart from the above-discussed processing strategy, another approach toward dataset segmentation has been explored. In this second approach, the datasets have been segmented into training and validation subsets in a subject-wise manner. This process ensures that data signatures of a particular subject are not present in both the training and validation sets, thereby ensuring a robust evaluation strategy of the proposed algorithm. This study only considers data with 8 s of data length. In addition, in order to study the impact of over-lapping contiguous windows on classification scores, three overlapping scenarios have been considered under this approach. The three scenarios pertain to 25, 50, and 75% overlapping of contiguous windows. For the different overlapping percentages, the number of windows generated can be expressed in terms of Equation (1).


(1) m=(n−r)/(k−r)

where, *m* = number of windows, *n* = total samples, *r* = overlap sample, and *k* = window sample.

### 2.2. Deep Learning Architecture for Classification

**Figure 3:**
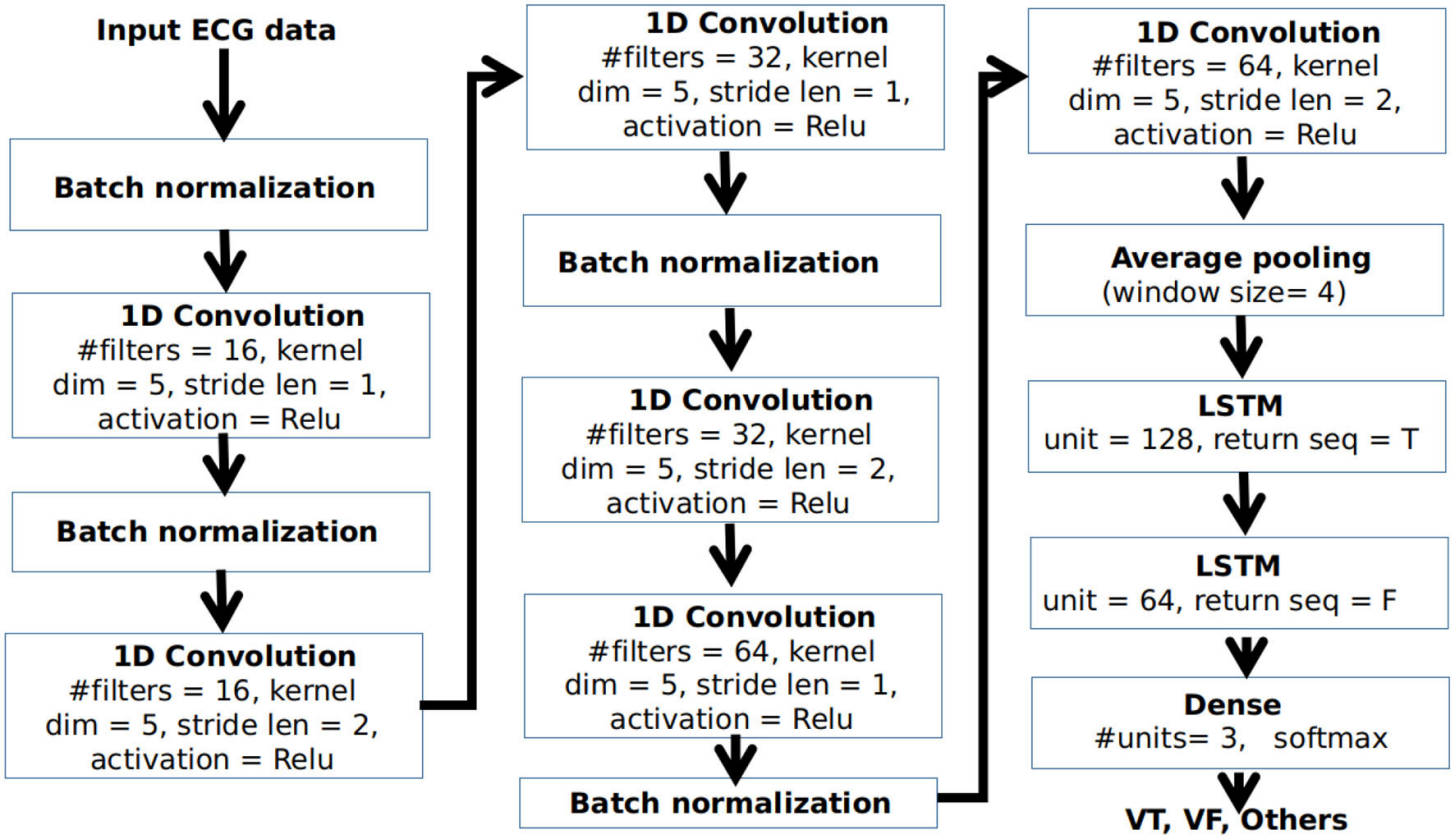
Block diagram of the proposed convolutional neural network-"full form of LSTM" (CNN-LSTM) architecture.

For classifying different arrhythmic rhythms, we propose a deep CNN-LSTM architecture for the classification of VT, VF, and other conditions from ECG. Here, other conditions can include any cardiac condition other than VT and VF, that do not require shock therapy. The block diagram of the proposed network architecture is shown in Figure 3.

The CNN-LSTM is a type of LSTM architecture specifically designed for sequence prediction problems for input data with spatial structure that can not be easily modeled with a vanilla LSTM. The architecture contains a series of CNN layers for the extraction of features from the input data which are then applied to an LSTM architecture for temporal modeling and prediction. As shown in Figure 3, input ECG data after pre-processing is applied to a series of 1D convolutional layers. For each convolutional layer, the kernel dimension is taken as 5. Batch normalization is applied at the end of the convolution operation for standardizing the inputs to a layer for each mini-batch. The number of filters in the base convolutional layers is selected as 16. As we go deeper, the number of filters in the convolutional layers are gradually increased by a factor of 2 to extract more detailed features. However, the dimension of the feature is reduced by applying the stride length of 2 in every alternate layer for doing the convolution operation. The neurons in the convolutional layer are activated using the non-linear Rectified linear unit (Relu) activation function. The output of the final convolutional layer is applied to an average-pooling layer having a window size of 4 to select a representative feature set at a reduced dimension. This is applied to a pair of LSTM layers having 128 and 64 units, respectively, followed by a dense layer having 3 neurons for classification of VT, VF, and other rhythms using a softmax activation function.

#### 2.2.1. Network Parameters

Selection of the optimum network hyper-parameters becomes a major challenge in designing a neural network architecture. In our architecture, we focused on a few parameters while designing the optimum architecture including, (1) number of filters in the base convolutional layer, (2) dimension of the filter kernel, (3) the stride length, and (4) number of hidden units in the LSTM layers. We opted for a randomized search as the hyper-parameter selection strategy, where the possible values of different hyper parameters are randomly selected from a pre-defined range to train and evaluate the network on a small representative dataset obtained from the CUDB database. The evaluation is done based on 5-fold cross-validation on the representative dataset. The combination of hyper-parameters producing the maximum median accuracy in the cross-validation approach is selected as the optimum combination for designing the network. The duration of instances in the representative dataset is considered as 4 s.

#### 2.2.2. Training of the Proposed Network

The proposed architecture is implemented in python 3.6.9 using TensorFlow 1.5. The platform where the network was trained contains an Intel Core i7 processor and 8 GB of primary memory. The mini-batch size is selected as 32. During training, the categorical cross-entropy loss of the network is minimized using an Adam optimizer with learning a rate of 0.005 and 300 epochs limit are used before stopping the training. The initial weights are set using Xavier initialization. In this process, the values are randomly assigned from a Gaussian distribution of zero mean and a finite variance $var=\frac{2}{n_{in}+n_{out}}$, where *n*_*in*_ and *n*_*out*_ are the number of input and output neurons in that layer, respectively. The bias terms are initialized by zeros.

### 2.3. Cardiac *in-silico* Model

The cardiac *in-silico* model is a computational model encompassing a 0D lumped hemodynamic model and a 3D volume conductor model enabling biophysical simulation. There is also electrophysiology (EP) block that can synthesize ECG template and is responsible for the initiation of cardiac contraction and pulsating behavior of heart chambers that drives the hemodynamic block. In this particular work, the ECG signal is used directly from the database or it can be the signal measured by sensing leads of WCD. If required, synthesized VF/VT ECG can also be generated using the proposed *in-silico* model (Mazumder et al., 2021).

#### 2.3.1. Hemodynamics Module

The Hemodynamic block consists of a four-chambered heart with lumped pulmonary and systemic circulations. The pressure variations across the cardiac chambers are modulated through time-varying compliance functions. Heart valves are modeled to replicate the functionality of each cardiac phase, capturing the pressure difference across the cardiac chambers to ensure unidirectional blood flow through the heart and maintain the pressure-volume dynamics. The model is also coupled with central nervous system modulation in terms of a baroreflex control, which regulates pressure autonomously through sympathetic and parasympathetic interaction of heart rate, contractility, and systemic vascular resistance, explained in detail in our prior works (Mazumder et al., 2019; Roy et al., 2021).

The dynamic equations to replicate the pressure dynamics of the model at various chambers and pulmonary and aortic arteries can be represented by state-space equations, depicting the flow variation due to resistance to blood flow from the vessel along with the compliance property of the chambers. As an example, the flow equation of the left ventricle is expressed in Equation (2).


(2) $$\begin{array}{ll} {\dot{P}}_{lv}= & \frac{1}{C_{lv}(t)}\left[U_{mi}\times \frac{P_{la}-P_{lv}}{R_{mi}}-U_{ao}\times \frac{P_{lv}-P_{sa}}{R_{ao}}-{\dot{C}}_{lv}(t)P_{lv}\right] \end{array}$$

Here *P*_*la*_, *P*_*lv*_, *P*_*sa*_ are the pressure variables in the left-atrium, left-ventricle, systemic arteries, respectively, *R*_*mi*_, *R*_*ao*_ are the valvular resistances across the mitral and aortic valve, *C*_*lv*_ is the left ventricle compliance. The symbols *U*_*mi*_, *U*_*ao*_ are the control functions to mimic the opening or closing of the respective cardiac valves. Pulsating action of the heart is driven by a compliance function, which determines the time-varying compliance of auricles and ventricles and brings about the pumping action of the heart, utilizing time and morphological metrics from ECG signal. This compliance adjustment is the most crucial part of this study as the effect of VF is modeled by decoupling atrium and ventricular compliance and then modulating the ventricular compliance to emulate the effect of VA.

**Figure 4:**
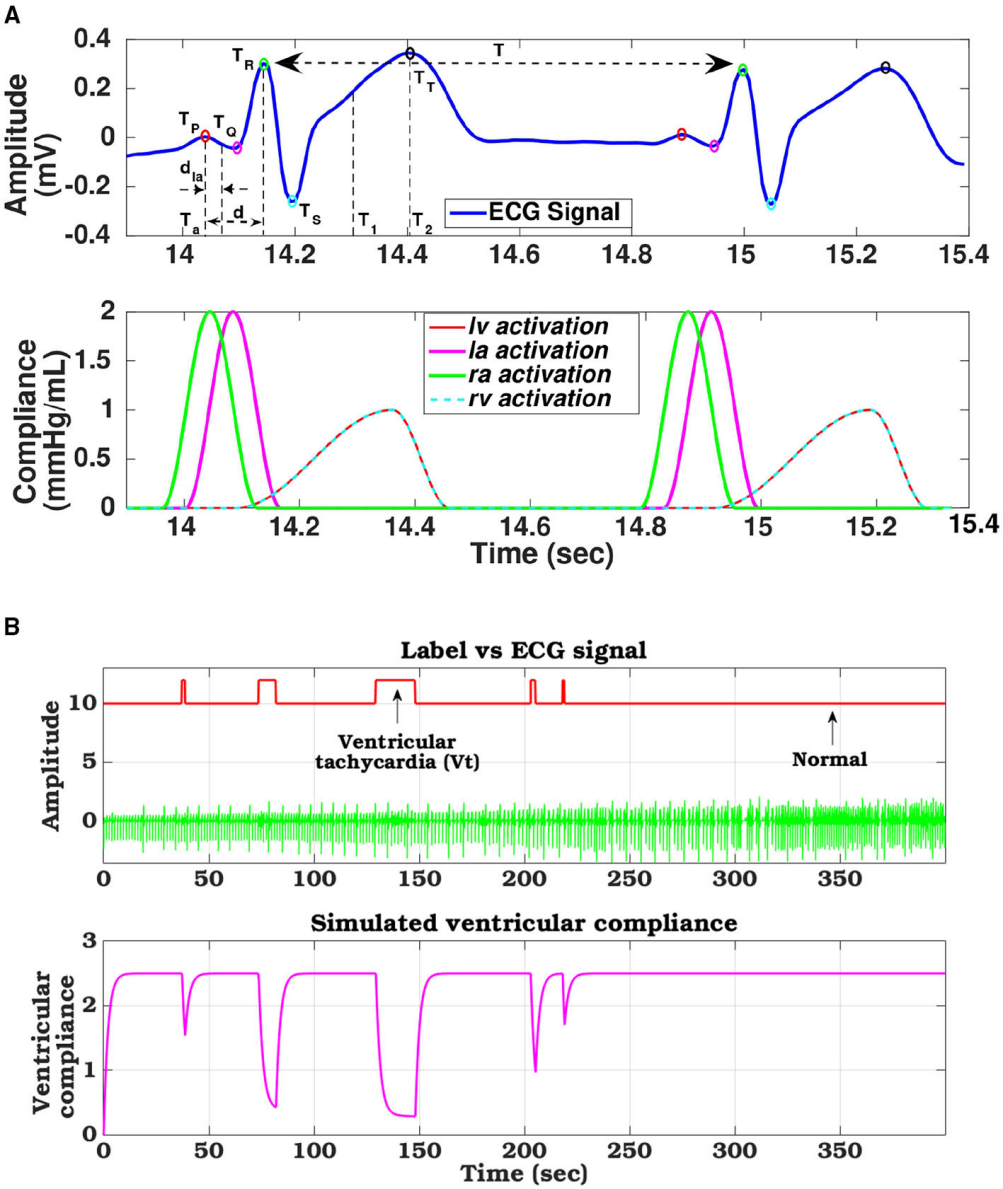
**(A)** ECG signal decomposed to its constituent components and phase matched cardiac chamber compliance functions, **(B)** Compliance variation of left ventricle tuned with arrhythmia ECG signal levels derived from the database.

In generic ECG signal, for one cardiac cycle, the characteristic cardiac electrical events like PQ (auricular depolarization), QRS (ventricular depolarization), ST duration (ventricular re-polarization), and R-R intervals are marked by a specific set of PQRST peaks whose amplitudes and time-instances can be represented as $\left[\begin{array}{ccccc} \left(P_{p},T_{p}\right) & \;\left(P_{q},T_{q}\right) & \;\left(P_{r},T_{r}\right) & \;\left(P_{s},T_{s}\right) & \;\left(P_{t},T_{t}\right) \end{array}\right]$ (Figure 4A). These electrical instances are encoded to modulate compliance function and timing information to control the synchronized operation of four heart chambers (Roy et al., 2021). Compliance function of the left ventricle can be modeled as follows:


(3) $$C_{i}(t)=C_{i}\times u_{v}(t-d),\;i\in \{lv,rv\}$$

(4) $$u_{v}(t)\;=\{\begin{array}{ll} 0.5-0.5\cos\left(\pi \frac{t}{T_{1}}\right), & 0\leq t<T_{1}\\ 0.5+0.5\cos\left(\pi \frac{t-T_{1}}{T_{2}-T_{1}}\right), & T_{1}\leq t<T_{2}\\ 0, & T_{2}\leq t<T \end{array}$$

where *u*_*v*_(*t*) is the activation function, and *d* = (*T*_*r*_ − *T*_*p*_) represents the delay in activation of ventricles from the right-atrium, *T*_1_ = (*T*_*r*_ + *T*_*t*_)/2 and *T*_2_ = *T*_*t*_ are the systolic and diastolic duration of the cardiac cycle (*T*), respectively. Similarly, compliance for the other chambers can also be modeled. The ventricular compliance (*C*_*i*_; ∀*i* ∈ {*lv, rv*}) are computed by the ratio between the R-peak and T-peak, expressed as $C_{i}=\frac{P_{r}}{P_{t}}$. Compliance functions estimated from ECG template for a healthy heart for all the 4 chambers are shown in Figure 4A.

Ventricular fibrillation is an abnormal heart rhythm, where irregular heart signals cause the ventricles to twitch uselessly. As a result, the cardiac elasticity across the ventricles increases, and hence, those cardiac chambers get stiff with decreased compliance (Arts et al., 2005). Subsequently, the heart does not pump blood to the rest of the body. To hemodynamically simulate this effect, the compliance function during the VF condition has been remodeled. Let us assume that $C_{\left(v,Nor\right)}^{i}\left(t\right)$, where, (*i* ∈ *RV,LV*) is the compliance across the ventricles (right and left) during normal conditions at the *t*^*th*^ time. When the VF episode starts, $C_{\left(v,Nor\right)}^{i}\left(t\right)$ starts decreasing. To model this effect analytically, we have formulated the following equation:


(5) $$C_{v}^{i}(t)=\{\begin{array}{ll} C_{v,Nor}^{i}\times exp(-\frac{t}{\tau }), & \text{if }VF=1\\ C_{v,Nor}^{i}, & \text{else } \end{array};\;\forall i\in \{RV,LV\}$$

where, *t* is the duration of the VF/VT episode, and τ defines the time constant of that VF episode. Thus, during the VF occurrence, the ventricular compliance starts decreasing exponentially. One such instance of remodeled ventricular compliance phase tuned with VF/VT occurrence is shown in the Figure 4B. The raw ECG signal along with the annotated labels, derived from the CUDB dataset is used as a reference to show the modulation of ventricular compliance with changes in ECG morphology and arrhythmic patterns.

#### 2.3.2. Biophysical Modeling

Understanding and replication of defibrillator behavior need reconstruction of torso-cardiac anatomy with bio-physically detailed realistic-geometry models. We have used an MRI scan of a 19-year-old healthy subject, obtained from a dataset developed by an Open Source software project of the SCI Institute's NIH/NIGMS CIBC Center (SCI, 2016) to create a 3D torso-cardiac model. Conductivity levels of various organs and tissues in the torso section, like the skin, skeletal muscles, fat, bones, lungs, spleen, liver, stomach, kidneys, and spinal cord are defined as per standard values reported in the literature (Lim et al., 2018). Finite element meshes are created in the 3D cardiac-torso model to help in solving the biophysical model associated with the application of external fibrillation. This is similar to forward electrophysiology, the only difference being that instead of using cardiac potential as the source model, defibrillator voltage is acting as the source. We have used monodomain equations to solve the biophysical model. Shocking electrodes are placed in the torso section for various possible configurations. Effect of an external voltage applied at the electrodes is captured through the modified torso and cardiac potential generation. The standard shocking configuration in WCD is *via* Apex-Posterior shock. In one of our previous works (Mazumder and Sinha, 2021), we compared three other shocking electrode configurations to obtain optimized defibrillation, expressed in terms of the critical mass hypothesis. In that analysis, Front-Back configuration resulted as the most optimized electrotherapy. In this work, we extend our previously designed defibrillator efficacy concept and focus on analyzing electrotherapy responses at various sub-locations in Apex-Posterior and Front-Back configurations.

The governing equation for biophysical simulation is the modified steady state electrical potential in an inhomogeneous volume conductor described by Laplace equation:


(6) ∇(σ∇ϕ)=0

where, σ is the conductivity tensor field and ϕ is the electric potential. This is subjected to two boundary conditions, Dirichlet boundary condition, defined as ϕ(*x,y,z*) ∣_Ω_*k*__ = *V*_*k*_ applied anywhere the electric potential is known (*V*_*k*_ is the known potential of electrode k, and Ω_*k*_ specifies the domain coincident with electrode k) and Neumann boundary condition, defined as $\frac{\partial \phi }{\partial n}{|}_{\Omega }=0$, applied on the surface of the object being simulated, not defined by Ω. In this implementation, we assume a linear and isotropic volume conductor model, with negligible capacitance and inductance, and applied the Galerkin finite element formulation with tri-linear interpolation (Colley et al., 2019). All the processes involved in a biophysical simulation like monodomain equation solving, mesh model generation, and their visualization were done using SCIRun software (Burton et al., 2011). For computing the biophysical equation, the torso model and the electrode model (defined over any place in Ω) are integrated into a computational mesh composed of hexahedral elements suitable for finite element modeling. Mesh created for the cardiac structure consists of 34,927 elements with 1,17,649 nodes, while the torso structure has 45,328 elements and 8,25,871 nodes, build around a lattice volume of 50 x 50 x 75 cm. SCIRun uses BioPSE modeling library and packages like TetGen for generating mesh structure (Stinstra et al., 2007). During simulations, boundary conditions are specified on all finite element nodes within the geometrical regions defined by the electrodes above. Electrodes are assigned a constant potential over their surface. For shocking electrodes (anode), the extracellular potential was fixed at the specified values to define the strength of the applied shock (500 V); for ground electrodes (cathode), the extracellular potential was defined to be 0 V throughout.

**Figure 5:**
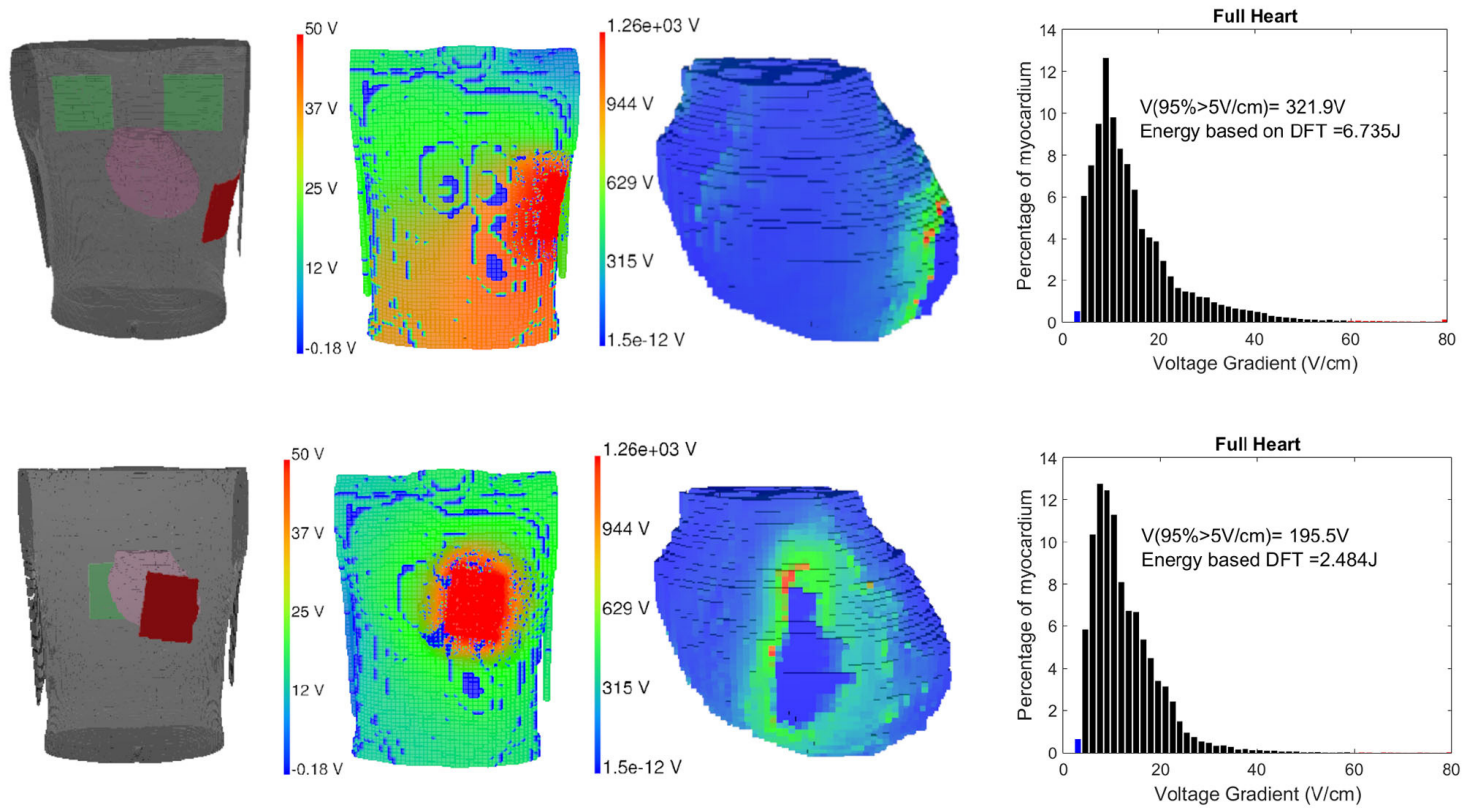
Left to right: Electrode location, torso potential distribution just after defibrillation, cardiac potential indicating complete myocardial de-polarization state and defibrillation threshold (DFT) histogram representation for Apex-Posterior **(upper)** and Front-Back configuration **(lower)**.

There are various theories to define defibrillation efficacy, but the underlying principle for all of them suggests that for fibrillation to be effective, the Defibrillation threshold voltage (DFT) value should be high enough to stop the fibrillation effect but lower than upper threshold level (ULV), that is capable of regenerating fibrillation mechanism through reentry (Karagueuzian and Chen, 2001). We have implemented the Critical point theorem (Zipes et al., 1975) which considers DFT value capable of changing at least 95% myocardial mass to a potential gradient of 5*V*/*cm* as a measure of complete defibrillation. After the potential distribution is solved using the finite element method, the gradients of the potential field are evaluated for the full thorax using tri-linear spatial derivatives and DFT values are computed. The DFT surrogate intrinsically obtains extracellular potential fields throughout the 3D volume of the myocardium making it inherently convenient for use in computational modeling studies in real-time (Morgan et al., 2009). Along with DFT, ventricular mass with voltage gradient distributions are also calculated. Higher voltage gradient (>30 V/cm) causes irreversible damage to the myocardium (Dosdall et al., 2010). Defibrillation energy is calculated using the formulation for energy type defibrillator, defined as $E=\frac{1}{2}CV^{2}$ where *C* = 130μ*F* and *V* is the required voltage DFT for the particular electrode configuration (Reek et al., 2017). In Figure 5, the electrode locations in the 3D volume model (cardiac-torso integrated), torso potential and cardiac potential just after defibrillation, and the myocardial voltage gradient for Apex-Posterior and Front-Back configurations are shown. Apex-Posterior is the default standard shocking electrode configuration used in WCD. Out of the two posterior electrodes, only one is functional at any given time. For our analysis, we have considered the right electrode as the shocking electrode. The cardiac potential reflects the state of complete cardiac depolarization, indicating effective defibrillation. The histogram representation of voltage gradient distribution gives an idea of the defibrillation pattern and efficacy.

#### 2.3.3. Defibrillation Evaluation

Defibrillation threshold value reaching the critical mass is capable of stopping the VA but the shock magnitude itself has sufficient energy to damage the myocardium. We calculate the ventricular mass with a voltage gradient >30, >45, and >60*V*/*cm*, to assess possible myocardial damage. A new measure combining DFT and myocardial damage is formulated using probabilistic distribution and weighted KL divergence (KLD) (Mazumder and Sinha, 2021). We define an ideal distribution of myocardial voltage gradient after defibrillation by combining two exponential functions, one rising and the other decaying in amplitude for below and above of 5*V*/*cm*, respectively. The distribution is defined such that the required critical mass defibrillation is achieved ideally around the 5 *V*/*cm* mark and the decay component diminishes for a value of the voltage gradient (*x*) ≥ 30*V/cm* (Zipes et al., 1975). Considering the modeled distribution as *M* and the defibrillation Voltage gradient distribution as *C*, the divergence or the information gain from *M* to *C* can be computed using KLD (Sekeh et al., 2013). Higher voltage gradient leads to greater myocardial damage, hence we have proposed the error measure reflecting the efficacy of the defibrillation (ED) using weighted KLD (WKLD) $D_{KL}^{w}$. Here, the weight (*W* = *x*) allows the regions with a higher myocardial gradient to be penalized more in the computation of the error measure (ED). Lower the measure, lower is the difference in entropy between M and C, making the actual defibrillation function closer to the modeled or ideal one. This difference can be considered as the error between these two distributions and provide an informative efficacy measure (ED) combining both DFT and myocardial damage information expressed as follows:


(7) $$WKLD(C)=D_{KL}^{w}(C||M)=\sum_{x=0}^{\infty }x.C(x)ln\frac{C(x)}{M(x)}$$

**Figure 6:**
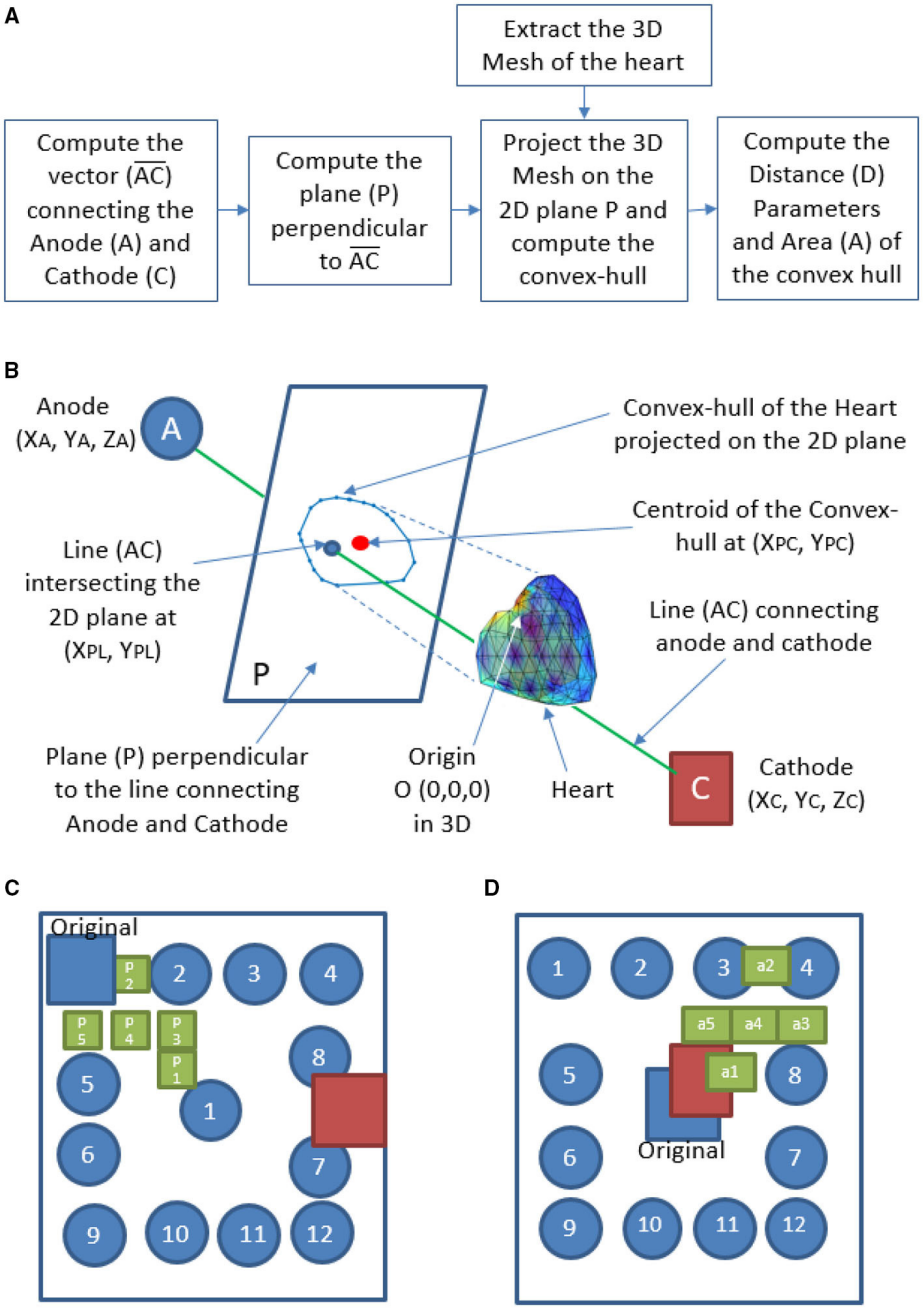
Calculation of metrics derived from the relative orientation of heart with respect to the WCD electrodes-**(A)** flow chart, **(B)** schematic representation, **(C)** electrode locations considered for Apex-Posterior, and **(D)** Front-Back configuration.

Defibrillation threshold, energy, and WKLD values are calculated for two standard shocking electrode orientations (Apex-Posterior and Front-Back) as well as in between various plausible subspaces of the specified configurations. WCD electrodes are all of similar shape and size (0.1 m × 0.1 m). For the Apex-Posterior configuration, the “apex” electrode acting as the cathode is positioned at the mid-axillary line at the level of the 5*th* intercostal space, apex coordinates being (0.1420, –0.074, and 0.0224) with respect to 3D world co-ordinate center (0,0,0). Here the representation is (*X*,*Y*, and *Z*), where *X* is along the medio-lateral, *Y* is along anterio-posterior and *Z* is along the vertical direction, the units are in meter (m). The center (0, 0, and 0) is taken as the center of torso at the transverse plane aligned center to the heart. Anode electrodes are placed under the right clavicle at the 4*th* intercostal level (−0.099, 0.0744, and 0.1225). The cathode electrode is placed on the left precordium, in front of the chest at coordinates (−0.0301, −0.0744, and 0.0225), and an anode is placed on the back behind the heart in between the scapulas (−0.0301, 0.0744, and 0.0225) for the Front-Back configuration (shown in Figure 5). For each of these configuration, we create a subspace of probable electrode locations to study the variation in defibrillation efficacy. We examine the variation of the defibrillation energy (E) and WKLD for each such electrode locations to find the location which minimizes the WKLD, while having a low and acceptable value of E. For a given configuration (Apex-Posterior or Front-Back), the variation in the position of the anode, leads to a different view point from which the electric field is propagated through the myocardium. Such change in the relative view point, with respect to the orientation of the heart, is quantified using certain distance and area metrics. In total, four metrics are defined namely, three distance metrics *D*1, *D*2, *D*3, and one area metric *A*1. The flow chart of the computation is given in Figure 6A. A schematic representation of the axes, planes, and projections used to define the metrics is shown in Figure 6B. *D*1 is the perpendicular distance from the origin of the world 3D coordinate “O” to line “AC” connecting the center of the anode and cathode. *D*2 is the distance between “O” and the center of the anode “A.” To compute the remaining metrics, the structure of the 3D heart is projected on the plane perpendicular to the line connecting the anode and cathode. The vector $\vec{AC}$ is computed using the centers of the anode (*X*_*A*_, *Y*_*A*_, *Z*_*A*_) and cathode (*X*_*C*_, *Y*_*C*_, *Z*_*C*_), which defined the view direction of the electrodes. Next, a projective transformation matrix (*T*_*O*_) is derived (given by Equation 8) to project any data point from the world 3D coordinate to a plane (*P*) perpendicular to the vector $\vec{AC}$.


(8) $$T_{O}=\left[\begin{array}{cccc} m_{11} & \;m_{12} & \;m_{13} & \;0\\ m_{21} & \;m_{22} & \;m_{23} & \;0\\ m_{31} & \;m_{32} & \;m_{33} & \;0\\ 0 & \;0 & \;0 & \;1 \end{array}\right]$$

The *m*_*ii*_ indicates the rotation and scaling related parameters and the entries in the last row and last column, except *m*_44_ are zeros indicating no translation and the scenario of orthographic projection (Hu et al., 2014). Recently, the orthographic projection was also used to analyze the structure of various parts of the human heart (Sherknies et al., 2003; Liu et al., 2019). We have considered such a projection to preserve the relative distances between a pair of points from the 3D space to the plane, which is required for the computation of the distance and area metrics on the plane P. A 3D mesh of the heart is created and then the *V* vertices of the mesh, with coordinates ($v_{xh}^{i},v_{yh}^{i},v_{zh}^{i}$), ∀1 ≤ *i* ≤ *V*, are projected to the 2d plane *P* using the transformation matrix *T*_*O*_ as shown in Equation (9).


(9) $$\left[\begin{array}{c} v_{xp}^{i}\\ v_{yp}^{i}\\ v_{zp}^{i}\\ 1 \end{array}\right]=T_{O}\times \left[\begin{array}{c} v_{xh}^{i}\\ v_{yh}^{i}\\ v_{zh}^{i}\\ 1 \end{array}\right]$$

The vertices of the 3D mesh are used as homogeneous form (${\left[v_{xh}^{i}v_{yh}^{i}v_{zh}^{i}\;1\right]}^{T}$), where *T* denotes the transpose. The projected coordinates ${\left[v_{xp}^{i}v_{yp}^{i}\right]}^{T}$ lies on the 2D plane *P* perpendicular to the view vector $\vec{AC}$. A convex-hull (*C*_*H*_) is derived using the 2D co-ordinates ${\left[v_{xp}^{i}\;v_{yp}^{i}\right]}^{T}$ on the plane *P*. The centroid of the *C*_*H*_ is computed as *X*_*PC*_, *Y*_*PC*_, and the line *AC* intersects the plane *P* (*atX*_*PL*_, *Y*_*PL*_) as shown in Figure 6B. The metric *D*3 is the distance between (*X*_*PL*_, *Y*_*PL*_) and *X*_*PC*_, *Y*_*PC*_, and the metric *A* is the area of the convex hull.

Along with the standard Apex-Posterior and Front-Back configuration, 12 similar orientations are recreated by placing the anode electrode in different locations and recomputing the mesh and biophysical simulations, keeping the cathode fixed. The top-most right location being (–0.1025, 0.0744, and 0.1025) and bottom left location being (0.1025,0.0744, and –0.1025). The other 10 electrodes are distributed in a symmetrical pattern with a 0.05 m gap in both “*X*” and “*Z*” axes. From the findings of these 12 new locations for each configuration, a finer distribution is studied by introducing 5 separate electrode locations in places translating to a better defibrillation index. The electrode sub-locations throughout the torso space are shown in Figures 6C,D.

## 3. Results

Initially, we present the results regarding the accuracy of detection of the shockable rhythms based on two datasets namely, CUDB and the VFDB. Then, the characteristics of the hemodynamics obtained from the cardiovascular simulation model, during both shockable and non-shockable segments, are given. Finally, results on the defibrillation metrics are presented for different electrode positions in two configurations namely, Apex-Posterior and Front-Back.

### 3.1. Detection of Shockable Rhythm

This sub-section details the performance of the proposed deep learning classifier in identifying VT, VF, and other classes on both CUDB and VFDB datasets based on a 5-fold cross-validation approach. The ECG measurements from all subjects, as available in the original dataset, are first segmented into small non-overlapping windows of equal length. Every single window is considered as an independent training or test instance for the classifier. The objective of this study is not only to measure the classification accuracy but also to estimate the optimum window length required for robust classification performance. Table 2 summarizes the classification performance of the proposed CNN-LSTM architecture on the CUDB and the VFDB datasets on different window lengths of 2, 4, 6, and 8 s. Here, we report the median classification performance in terms of precision, recall, and F1-score of detecting various target classes across 5-fold cross-validation.

**Table 2:** Classification performance of the proposed deep learning classifier on CUDB and VFDB dataset (*P* = precision, *R* = recall, *F*1 = *F*1 score).

	**2 s**			**4 s**			**6 s**			**8 s**		
**Target** **label**	**P**	**R**	***F*1**	**P**	**R**	***F*1**	**P**	**R**	***F*1**	**P**	**R**	***F*1**
VF (CUDB)	0.96	0.92	0.94	0.94	0.99	0.97	0.97	0.96	0.96	0.97	0.98	0.98
VT (CUDB)	0.89	0.66	0.76	0.67	0.47	0.55	0.50	0.83	0.62	1.00	0.69	0.82
Others (CUDB)	0.98	0.99	0.99	1.00	0.99	0.99	1.00	0.99	0.99	0.99	1.00	1.00
VF (VFDB)	0.73	0.81	0.77	0.75	0.85	0.80	0.85	0.70	0.77	0.82	0.78	0.80
VT (VFDB)	0.80	0.69	0.74	0.78	0.70	0.74	0.70	0.83	0.76	0.74	0.81	0.77
Others (VFDB)	0.99	0.99	0.99	1.00	0.99	0.99	0.99	1.00	0.99	1.00	0.99	1.00

Precision, recall, and F1-score are popularly used for measuring the performance of a classifier. In theory, precision measures the number of correct positive predictions, and recall measures the number of correct positive predictions made out of all positive predictions that could have been made by the classifier. For a multi-class classifier, these two are defined for every target class in terms of true positive (TP), true negative (TN), false positive (FP), and false negative (FN), across all classes in one vs. all method:


(10) $$precision=\frac{TP}{TP+FP}\;recall=\frac{TP}{TP+FN}$$

F1-score is a method of measuring the classification accuracy based on the combined effect of precision and recall. Mathematically, it measures the harmonic mean of precision and recall as follows:


(11) $$F1=2\frac{\left(precision*recall\right)}{precison+recall}$$

As mentioned in Section 2.1, both our target datasets are largely imbalanced. Among the 3 different classes (VT, VF, and other), the instances corresponding to the other conditions occupy the major portion of both CUDB and VFDB datasets, whereas the number of instances related to VT is the least in number. It can be observed from Table 2 that the overall F1-score of detecting various classes tends to improve with the increased window-length and the optimum performance is achieved at a window-length of 8 s. It is to note that the overall signal quality of the CUDB datasets is better than the VFDB dataset, which contains many noisy instances where the classifier does not yield reliable performance. Hence, the proposed classifier produces a better classification accuracy on the CUDB dataset compared to VFDB.

Table 2 summarizes the classification performance of our proposed approach on the CUDB and the VFDB databases. Similar to the existing approaches in the literature, the classification approach is evaluated by applying cross-validation on the entire dataset and the accuracy is reported against a fixed window length. However, this approach does not reveal the utility of the classifier in detecting the onset of a VT or a VF event on a long data stream recorded from a subject. Hence, in the second part of our experiment, we perform a detailed subject-wise analysis.

Typically, a shock is applied within 32 s of detecting a VT or a VF event. Hence, in this stage we evaluate our classification performance on every 32 s long data segment. The decision window is fixed at 8 s and a final decision corresponding to a 32 s data stream is made based on majority voting on the prediction labels on the continuous 8 s long windows within the 32 s long data stream. For the subject-wise analysis, we completely separate the train and test subjects on both datasets considered in this article. About 80% of all subjects form the training set and the remaining 20% of the subjects form the test set. The deep learning model is retrained on the new training set and is individually applied on every test subject for prediction. Different hyper-parameters of the neural networks are kept unchanged. To analyze the detection accuracy of the onset of an event, we applied overlapping on the successive 8 s-long windows, in a 32 s long data-stream. Table 3 shows the impact of overlapping in classification performance based on non-overlapping, 25, 50, and 75% overlapping. Depending upon the amount of overlapping, a 32 s long data stream contains 4, 5, 7, and 13 data windows.

**Table 3:** Subject-Wise classification performance overlapping windows of 8 s on CUDB and VFDB dataset (*P* = precision, *R* = recall, *F*1 = *F*1 score).

**Overlappingwindow**	**0**%****			**25**%****			**50**%****			**75**%****		
**Target label**	**P**	**R**	***F*1**	**P**	**R**	***F*1**	**P**	**R**	***F*1**	**P**	**R**	***F*1**
VF (CUDB)	0.93	0.96	0.94	0.98	0.98	0.98	0.98	1.00	0.99	0.99	0.99	0.99
VT (CUDB)	0.95	0.60	0.74	0.98	0.75	0.85	0.98	0.90	0.94	0.97	0.92	0.90
Others (CUDB)	0.98	0.98	0.98	1.00	0.99	0.99	1.00	1.00	1.00	1.00	1.00	1.00
VF (VFDB)	0.82	0.78	0.80	0.90	0.82	0.86	0.97	0.90	0.93	0.99	0.90	0.93
VT (VFDB)	0.71	0.80	0.75	0.77	0.89	0.83	0.88	0.94	0.91	0.80	0.94	0.85
Others (VFDB)	1.00	0.98	0.99	1.00	1.00	1.00	1.00	1.00	1.00	1.00	1.00	1.00

### 3.2. Hemodynamic Parameter Variations

**Figure 7:**
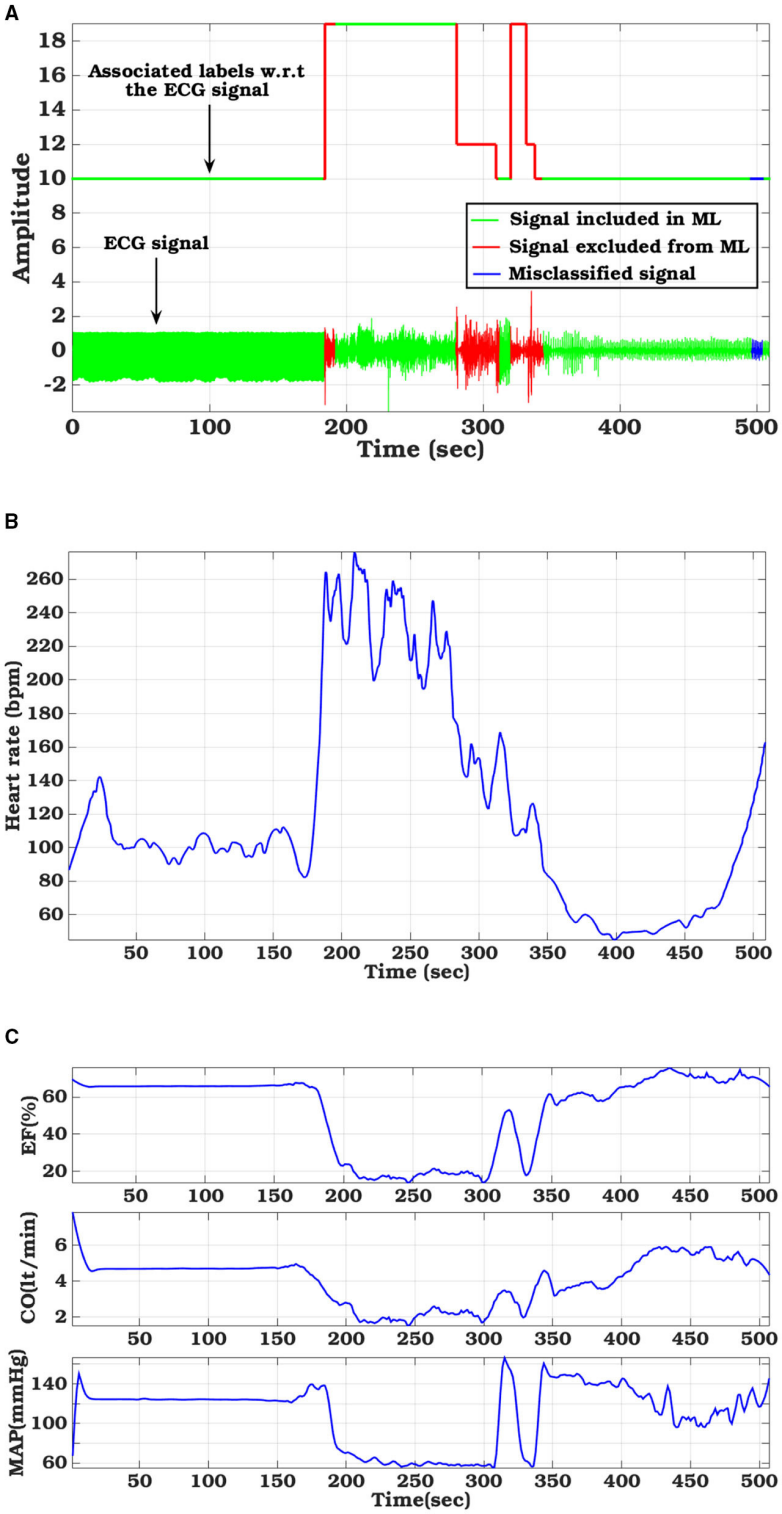
Hemodynamic parameter variations tuned with sample ECG signal from CUDB database: **(A)** ECG signal with ground truth label and classifier derived labels, **(B)** Variation in Heart Rate, **(C)** left-ventricular functional metrics (Ejection fraction, Cardiac output, and Mean arterial pressure).

The hemodynamic module takes ECG signal as the driving parameter and based on the morphological variation, apparent during VF/VT, adjusts the left ventricular compliance, as explained in Section 2.3.1. Figure 7A shows particular instances of ECG waveform variation along with the ground truth label and the performance of our proposed classifier. Heart rate (Figure 7B), calculated from the ECG signal is also displayed. Based on the detected VF/VT regions, compliance is modulated. Left ventricle compliance, as shown in Equation (2), dictates the pressure-flow dynamics of the systemic circulation. As *C*_*lv*_, heart rate, and flow parameters vary due to change in cardiac contractility, there is a marked effect on inherent cardiac parameters like ejection fraction (EF), cardiac output (CO), mean arterial pressure (MAP), etc. (Figure 7C). These parameters are of paramount medical importance in analyzing cardiac function. LVEF is the most important factor in stratifying SCD. EF, as captured from the computational model, shows a marked reduction during VF/VT period, which if left uncorrected will lead to SCD. In the dataset used, the VF episodes were occurring randomly for a short duration along with normal sinus rhythm. The hemodynamic module adapts to these changes in conduction dynamics and computes left ventricle information in real-time, without any additional delay. Along with EF, CO and MAP also follow pathological trends observed during VF/VT episodes. MAP calculated takes into account the change in cardiac contractility, heart rate variation, and also change in systemic resistance, regulated through a baroreflex mechanism. Hence, these observations are not just reflections of the initiation of pathological conditions, some level of modulation offered by the Central nervous system in an attempt to regularize the hemodynamic turbulence is also encoded in it. This trend is especially evident in post-VF episodes, where the ground truth label is normal but there are fluctuations in MAP trying to maintain the homeostasis.

### 3.3. Defibrillation Metrics

The results provided for defibrillation efficacy computation are an extension of our previously published work (Mazumder and Sinha, 2021), where we compared four different shock electrode configurations, naming Apex-Anterior, Apex-Posterior, Side-Side, and Front-Back, and found that the unconventional Front-Back configuration yielded better defibrillation efficacy compared to the other configurations. In this work, we extend the concept of varying the electrode location in a sub-plane around the Apex-Posterior and Front-Back configuration to analyze the effect of inter-electrode distance and effect of electrode location in overall defibrillation efficacy. Table 4 tabulates 4 different distance metric vectors along defibrillation efficacy metrics in terms of WKLD and Energy for 13 locations in Apex-Posterior and 13 Front-Back configurations. The configurations are shown in Figures 6B,C. It is interesting to note that apart from the original standard location, there are other locations that reports even lower WKLD and energy value, pointing to location where fibrillation is more efficient. For Apex-Posterior configuration, locations 2, 5 coinciding with the upper right quadrant, and for Front-Back, locations 3, 4, and 8, coinciding with the upper left quadrant shows the most decreased WKLD and DFT energy trend. Similarly, electrodes lying in lower torso quadrants show increased defibrillation energy. For defibrillation, an optimal position is defined as the position of anode and cathodes that allow maximum current path. Current conduction, apart from the geometry of electrodes and voltage applied, also largely depends on the trans-thoracic Impedance (TTI) and intra-thoracic impedance. TTI is dependent on numerous factors like torso geometry, respiration rate and phase, electrode size, etc. and varies from person to person while intra-thoracic impedance, dictated by thoracic organs and tissue may remain fairly constant in normal physiology but gets changed drastically in cardiac conditions like heart failure (Wang, 2007). As we are considering only a single subject scan data, TTI is assumed to be constant. So the current conduction path is mostly a function of a distance vector and tissue conductivity in the thoracic chambers. Out of the four distance metrics defined, D1 and D3 values are less in the Front-Back configuration as compared to Apex-Posterior. Metric A1 and D2 do not show much variations in changing the electrode locations. It is interesting to note that as the electrodes are organized throughout the torso in a geometric fashion, electrodes in the upper and lower quadrants have fairly equivalent distance metrics calculated from the center of the heart, yet, defibrillation energy required in some quadrants is comparatively higher than others. This is mainly due to the high conductivity indices of tissues in the upper torso, decreasing the impedance in the current pathway, and paving the way for efficient defibrillation. In Table 5, a more concentrated torso area is analyzed. Based on the deduction from Table 4 on the possible optimal electrode location, 5 new electrode locations both for Apex-Posterior and Front-Back configurations are analyzed. The distance metrics, DFT voltage, defibrillation energy, WKLD value, and Percentage of myocardial volume (*%myo*) above critical gradient capable of causing myocardial damage are shown.

**Table 4:** Defibrillation efficacy analysis on varying electrode location (loc-location, O-original, E-Energy, J-Joule, the units of *D*1, *D*2, *andD*3 are meter (*m*) and *A*1 is *m*^2^).

	**Apex-Posterior**						**Front-Back**					
**loc**	**D1**	**D2**	**D3**	**A1**	**WKLD**	**E(J)**	**D1**	**D2**	**D3**	**A1**	**WKLD**	**E(J)**
O	0.096	0.099	0.053	0.009	11.085	6.735	0.052	0.030	0.057	0.009	4.854	2.483
1	0.084	0.050	0.062	0.007	24.256	5.283	0.083	0.103	0.069	0.010	5.982	11.906
2	0.066	0.053	0.056	0.009	7.578	5.847	0.077	0.053	0.068	0.010	5.827	5.595
3	0.088	0.053	0.064	0.007	19.168	4.800	0.040	0.053	0.023	0.008	4.664	2.471
4	0.137	0.103	0.060	0.008	29.414	13.121	0.052	0.103	0.033	0.009	4.443	4.882
5	0.098	0.103	0.054	0.010	8.274	9.399	0.083	0.103	0.069	0.010	6.583	9.615
6	0.098	0.103	0.054	0.010	46.953	25.437	0.083	0.103	0.069	0.010	5.982	11.906
7	0.137	0.103	0.060	0.008	126.765	43.836	0.052	0.103	0.033	0.009	20.696	11.143
8	0.137	0.103	0.060	0.008	55.518	16.087	0.052	0.103	0.033	0.009	3.597	3.530
9	0.098	0.103	0.054	0.010	57.113	36.115	0.083	0.103	0.069	0.010	15.478	18.736
10	0.066	0.053	0.056	0.009	60.734	29.313	0.077	0.053	0.068	0.010	16.600	14.800
11	0.088	0.053	0.064	0.007	85.297	33.501	0.040	0.053	0.023	0.008	20.777	13.214
12	0.137	0.103	0.060	0.008	92.375	49.465	0.052	0.103	0.033	0.009	21.686	16.757

**Table 5:** Defibrillation efficacy analysis on upper torso concentrated sections, M1 = 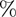
*myo* > 30*V*/*cm*, M2 = 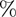
*myo* > 45*V*/*cm*, M3 = 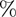
*myo* > 60*V*/*cm*, (loc-location, O-original, E-Energy, J-Joule, the units of *D*1, *D*2, *andD*3 are meter (*m*) and *A*1 is *m*^2^).

**loc**	**D1**	**D2**	**D3**	**A1**	**DFT(V)**	**E(J)**	**WKLD**	**M1**	**M2**	**M3**
p1	0.066	0.052	0.056	0.009	268.5	4.687	8.099	6.092	0.868	0.129
p2	0.042	0.030	0.067	0.008	269.9	4.734	10.535	6.568	1.007	0.138
p3	0.066	0.052	0.056	0.009	272.0	4.809	6.204	4.756	0.564	0.102
p4	0.083	0.075	0.053	0.009	326.9	6.944	5.340	4.584	0.576	0.111
p5	0.098	0.102	0.053	0.009	387.3	9.750	5.721	5.109	0.612	0.102
a1	0.040	0.052	0.022	0.008	159.0	1.642	3.260	1.556	0.252	0.0667
a2	0.046	0.075	0.029	0.008	205.3	2.740	4.792	3.177	0.646	0.182
a3	0.051	0.102	0.032	0.009	255.2	4.234	3.745	2.232	0.611	0.249
a4	0.046	0.075	0.029	0.008	184.4	2.209	4.095	2.643	0.405	0.118
a5	0.040	0.052	0.022	0.008	173.1	1.946	3.798	2.174	0.342	0.104

## 4. Discussion

In this article, we present a computational pipeline integrating shockable rhythm detection and shock voltage field optimization for evaluation, testing, and personalization of a WCD design and operation.

In general, machine learning classifiers like an SVM or a random forest can be successfully deployed to predict cardiac events, where the clinical markers/ features are well-known and are relatively simple to compute from the ECG signals (Figuera et al., 2016). However, deep learning approaches are typically preferred in large scale analysis where the disease-specific markers are not very easy to compute in terms of numeric features to train a classifier. A deep learning approach can also deal with the internal noise present in the signal. Both CNN and LSTM based deep architectures have been successfully used in prior literature (Silva et al., 2019). Finding the optimum window length for decision making is an important parameter in biomedical classification problems. In general, a shorter window is preferred due to low latency in inference. However, a small window may not always contain the discriminating markers for accurate decision making. On the other hand, a longer window length may ensure the presence of a discriminating marker. However, there remains a risk of latency in inference which may delay in applying the shock. We evaluated classifier performance on varying the window length from very small windows of 2 s to larger windows of 8 s. As tabulated in Table 2, there is a trend of improved precision, recall, and F1 score for VF, VT, and all other rhythms were grouped as non-shockable with the increase in window length. Our proposed CNN-LSTM architecture achieves a sensitivity of 96.10%, specificity of 98.34% for shockable rhythms (VF and VT) detection on a very small window size of 2 s for CUDB data and sensitivity of 94.68%, specificity of 92.77% for the VFDB dataset. For 8-s window size, which is the standard size reported in many prior arts, our algorithm attains sensitivity of 99.21%, specificity of 99.68% for the CUDB dataset and sensitivity of 98.56%, specificity of 99.08% for the VFDB dataset. As per guidelines established by the American heart association (AHA) (Kerber et al., 1997), a sensitivity (Se) higher than 90% for shockable rhythms, and specificity (Sp) higher than 95% for non-shockable rhythms is the benchmark for WCD detection algorithms and our proposed method exceeds the benchmark requirement. Also while comparing with the state-of-the-art, as tabulated in Table 6, our sensitivity-specificity values are closely comparable to the highest accuracy reported by Jeon et al. (2020) for WCD applications. Although our reported accuracy is fractionally lower, it is important to note that apart from 8 s standard window-based classification, we have also implemented an overlapping window-based detection that actually spans over 32 s long data that may contain up to 13 windows depending upon the amount of overlapping.

**Table 6:** Comparison of existing algorithms for detection of shockable rhythms.

**Reference**	**Brief approach**	**Dataset used**	**Accuracy reported**
Figuera et al. (2016)	An ML-algorithms with built-in feature selection capabilities were used to determine the optimal feature subsets for classification. Patient-wise bootstrap techniques were used to evaluate algorithm performance on public database	Validated on the VFDB and the CUDB datasets	Sensitivity = 96.6%, Specificity = 98.8%
Kwon et al. (2018)	The authors proposed an embedded microcontroller where an ECG sensor is used to capture, filter and process data, run a real-time VF detection algorithms developed a VF detection algorithm, via Time Delay (TD), based on phase space reconstruction.	Open access MIT-BIH dataset	Sensitivity = 96.56%, Specificity = 81.53%
Krasteva et al. (2020)	A deep convolutional network was proposed and studied on Holter ECG recordings for detection of shockable and non-shockable rhythms. The impact of various network hyper-parameter tuning was reported	The data used in the study contains a wide variety of non-shockable and shockable rhythms from two sources: public Holter ECG databases from continuously monitored patients with ventricular arrhythmias, and OHCA databases recorded by AEDs from patients in cardiac arrest.	For analysis on short windows (2 s): Sensitivity 97.6% =, Specificity = 98.7%. For analysis on long windows (5 s) : Sensitivity = 99.6 % Specificity = 99.4 %
Jeon et al. (2020)	A deep architecture comprising convolutional layers and recurrent networks for classification of ECG beats. Furthermore, a lightweight model is proposed with fused RNN for speeding up the prediction time on central processing units (CPUs)	The authors used 48 ECGs from the open access MIT-BIH Arrhythmia Database, and 76 ECGs were collected with S-Patch devices developed by Samsung SDS	For the baseline model: Sensitivity = 99.86%, Specificity = 98.31% for the light-weight model: Sensitivity = 99.92%, Specificity = 99.11%
Our proposed approach	A CNN-LSTM architecture is proposed for classification of VF, VT and other rhythms from ECG	The approach is evaluated on CUDB and VFDB datasets	Detection rate of shockable rhythms (VF and VT) on CUDB: very small windows (2 s) Sensitivity = 96.10%, Specificity = 98.34% for large windows (8 s) Sensitivity = 99.21%, Specificity = 99.68% Detection rate of shockable rhythms (VF and VT) on VFDB: very small windows (2 s) Sensitivity = 94.68%, Specificity = 92.77% for large windows (8 s) Sensitivity = 98.56%, Specificity = 99.08%

Table 3 shows the classification performance on the ECG data-stream obtained on individual test subjects. Here, the training and the test data were created based on different subjects. The decision was made by combining multiple 8 s long windows in a 32 s time frame. Just like any other time signal, an ECG data-stream is not entirely stationary. Breaking a 32 s long block into multiple non-overlapping windows may cause information loss at the junction of two consecutive windows. Hence, we analyse the impact of overlapping windows by a applying various percentage of overlapping starting with non-overlapping to 25, 50, and 75% of overlapping. The number of 8 s long windows increases with the increased overlapping percentage which is able to capture more detailed features from the data stream. Table 3 clearly indicates that there is a positive impact on classifier accuracy due to overlapping. Overall classification performance in terms of precision, recall, and F1-score significantly improves over the non-overlapping scenario and reaches the optimum performance when a 50% overlapping is applied between successive windows.

A completely novel aspect of our proposed computational pipeline is the capability of generating hemodynamic parameters during VA. SCD though initiated by different causes is ultimately governed by the left ventricle EF (Sun et al., 2014). ICD/WCD requirement stratification is also modulated based on left ventricle functions (Arts et al., 2005). As such, only understanding the electrical aspects of cardiac functioning through arrhythmia propagation, without giving due importance to its mechanical functioning, results in a partial understanding of the disease etiology and defibrillation response. The proposed *in-silico* cardiac model captures the flow-pressure-volume relationship for each cardiac chamber and for all cardiac phases, thus providing a holistic understanding of the pathophysiological changes occurring as VF/VT initiates, propagates, and subsequently gets terminated naturally or through the application of shock. As the hemodynamic module is controlled *via* ECG signals (either simulated or captured in real-time or used from database), real-time phase matched comparative hemodynamic metrics like cardiac output, mean arterial pressure, cardiac compliance, ejection fraction, etc. can be studied with ECG signal variations due to arrhythmia or any other cardiac disease that changes the ECG morphology, like myocardial ischemia. Another important rationale for introducing the hemodynamic module is evident in Figure 7. The ECG signals may be misclassified at several small window locations, however, this has no impact on the outcome of hemodynamic variables as the hemodynamic parameters are not dependent on the classified signal annotations but reflect the true physiological changes during arrhythmic events. The cardiac compliance value gets modulated through heart rate extracted after ECG processing along with physiologically matched mathematical derivations of systemic and pulmonary resistance, aortic and chamber compliance etc. So even if the classification algorithm fails for any particular window, by judging the hemodynamic parameter variations, initiation of VA can be well speculated and analyzed offline.

We have tabulated some common hemodynamic parameters like CO, EF, MAP, end-systolic pressure volume ratio (ESPVR), and end-diastolic pressure volume ratio (EDPVR) for shockable and non-shockable rhythms utilizing labels and ECG signals from CUDB and VFDB datasets. As indicated in Table 7, there is a marked difference between VA hemodynamic parameters compared to other non fatal group. ‘Other’class compiled from VFDB and CUDB datasets are not healthy but agglomeration of different supra-ventricular, normal, atrial fibrillation type rhythm grouped as non-shockable. CO, indicative of the volume of blood pumped by the heart in a cardiac cycle, gets heavily reduced during VA, indicating LV failure. EF also gets lowered to a dangerous level indicating impaired LV functionality and subsequent heart failure, if left untreated. The MAP also drops significantly due to low CO. ESPVR is commonly used as a marker for cardiac contractility (Yaxin et al., 2017) and the tabulated value clearly shows the reduction in LV contractility under VA conditions. Similarly, EDPVR is a marker for chamber compliance (reciprocal relation) and is used to judge ventricular stiffness (Yaxin et al., 2017), which during our simulation, also followed a medically correlated trend. The hemodynamic insights not only provides a better understanding of the disease progression but also provide an idea about the operable timeline, to take necessary corrective action in case of heart failure trends. In real time operation of WCD, implementing such a hemodynamic module might not be practical in the embedded circuit used for arrhythmia detection. However, a cloud based implementation of such modules could aid physicians better, in assessing overall cardiac functionality during VF/VT episodes and/or other arrhythmic episodes and may help in generating revised treatment plans with a more subject-specific personalized focus.

**Table 7:** Hemodynamic parameter variation for shockable and non-shockable pathological conditions.

**Parameters**	**Shockable (VF/VT)**	**Non-shockable**
CO (lt/min)	2 ± 0.5	4.5 ± 1.2
EF (%)	25 ± 7.5	60 ± 5
MAP (mmHG)	60 ± 15	118.9 ± 20
ESPVR	0.36 ± 0.32	2.5 ± 0.5
EDPVR	0.4 ± 0.29	0.16 ± 0.04

In Table 4, where we tabulate the variations in electrode location and corresponding defibrillation metric, apart from the trend established in terms of optimized electrode positioning, additional important insights can be inferred. For both Front-Back and Apex-Posterior configuration in various sub spacing, the minimum energy requiring location is not the location that reports the minimum WKLD value. As WKLD integrates both DFT voltage and myocardial damage probability, it becomes quite evident that lower defibrillation energy does not necessarily suffice to minimum cardiac tissue damage. Overall, in all possible location variations, the Front-Back configuration results in better efficacy. Judging by the WKLD value, the most optimal location is “location 8” in Front-Back (FB) configuration and location 2 in Apex-Posterior (AP) configuration, while the least effective location is location 12 and location 7 for Front-Back and Apex-Posterior, respectively. The DFT voltage and % myocardium >45*V*/*cm* and >60*V/cm* for location 8 (in FB) and 2 (in AP) are 233V, 0.46, 0.191 and 299.9V, 0.72, 0.12, respectively, while for location 12 (in FB) and 7 (in AP), the respective metric values are 507.7V, 1.884, 0.5189 and 821.2, 19.60, 12.67. As observed, the far-away electrode locations require excessive DFT voltage, associated with a greater extent of myocardial damage. The observations from these metrics can indicate locations to avoid while placing electrodes and then can guide areas where optimal defibrillation efficacy can be expected.

**Figure 8:**
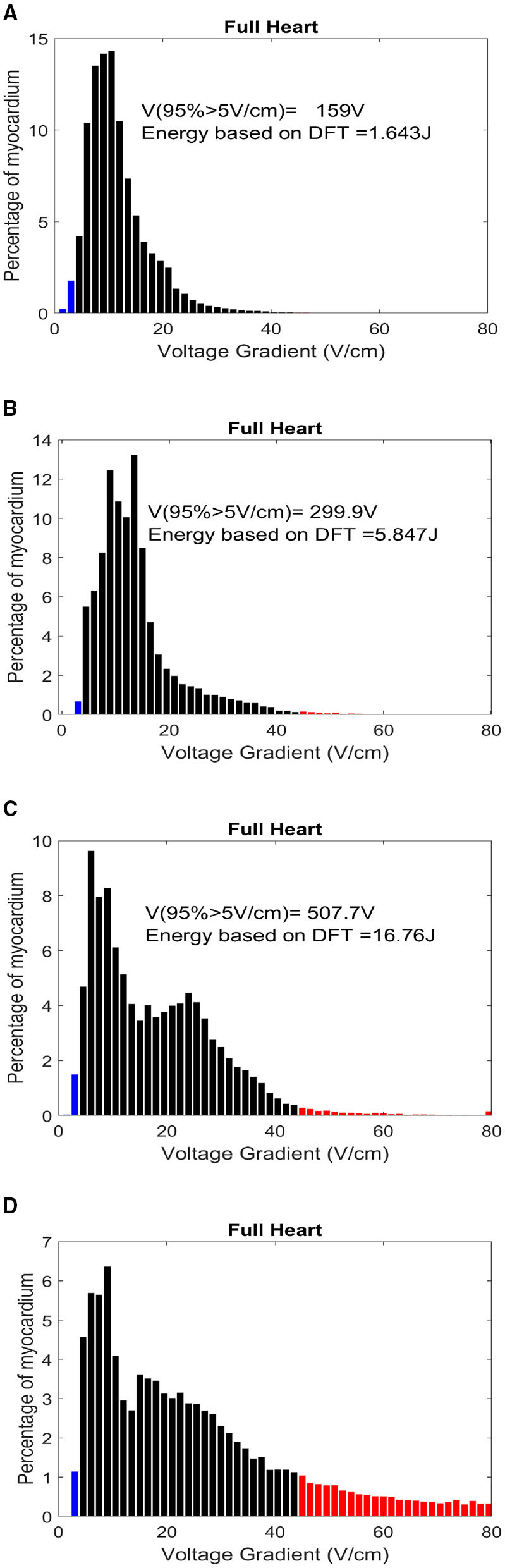
Histogram distribution of myocardial potential gradient for location **(A)** a1-FB, **(B)** 2-AP, **(C)** 12-FB, and **(D)** 7-AP. The red zones indicate a potential gradient harmful enough to create permanent myocardial damage.

In Table 5, the myocardial voltage gradient values are also generated to provide an indication of the relation between DFT voltage, energy, and distance metric in the specific torso area where optimal defibrillation pattern is expected, as deduced from Table 4. Location p4 in Apex-Posterior and a1 in Front-Back provided the least WKLD value and negligible probability of myocardial damage. Judging 18 locations each for both the configuration, Location 2 and a1 provide the best outcome in Apex-Posterior and Front-Back configurations, respectively. Figure 8 represents the myocardial voltage gradient histogram for location a1 (in FB), 2 (in AP) and 12 (in FB), 7 (in AP) as two best and two least desired electrode configurations, respectively. In the concentrated areas, situated in the upper thorax, the intra-thoracic conductivity parameters for both Apex-Posterior configuration as well as Front-Back configurations are fairly constant, the current path has to navigate mostly through the skin, skeletal structure, and lungs region. However, due to variation in the anode location, metric D1 and D3 are relatively shorter in Front-Back as compared to Apex-Posterior configurations, yielding a better current pathway and effective defibrillation.

Use of computational pipeline for shock distribution analysis and deriving metrics that can be incorporated in WCD vest and shock generating circuit for optimized defibrillation is a unique solution aimed at providing WCD shock efficacy validation and personalization. Such concepts have not been proposed earlier for WCD and have the potential to enhance conventional WCD functioning.

In this article, we have integrated two different aspects of WCD working in a biophysical computational framework for better understanding and validation of WCD performance both in terms of arrhythmia detection and shock efficacy computation through the DFT principle. While the hemodynamic module of the cardiac *in-silico* model provides additional insights into pathophysiological changes in cardiac functionality during arrhythmic episodes, the 3D volume conductor cardiac model and FE analysis with changeable electrode configuration provided an understanding of the defibrillator efficacy parameter variation with change in shocking electrode configuration and location. This is particularly useful for obese patients or pediatric users where the use of standard configuration may provide successful defibrillation but at the cost of higher myocardial damage. As our proposed model incorporates a monodomain modeling approach rather than a bi-domain, the realistic myocardial tissue behavior during defibrillation is not captured. However, as we do not intend to calculate absolute defibrillation response in myocardial tissue but aim to use the platform to provide an estimate by which the intra-thoracic field strength over the myocardium can be compared given differing electrode configurations, the computationally less extensive monodomain model is suitable. A particular drawback of this study is that the defibrillation efficacy simulation is based on single subject data and MRI data for multiple subjects with varying torso geometry would help to consolidate the electrode location variations observed. In future, for the classification of shockable rhythm, we would integrate the proposed algorithm in an embedded platform to make it suitable for real-time applications.

## 5. Conclusion

In this article, we present a computational pipeline for WCD performance validation, both in terms of shockable arrhythmia classification and optimal electrotherapy generation. We also derived some useful insights regarding the physiological changes in cardiac hemodynamics during Ventricular arrhythmic patterns leading to compromised LV functions. In the classification domain, our proposed CNN-LSTM architecture detection accuracy surpassed AHA recommended accuracy. The inclusion of the novel overlapping window approach guarantees a minimum loss of vital information in between detection windows, increasing the reliability of detection.

Cardiac defibrillators are lifesaving therapeutic devices with potentially harmful capacity if not tuned properly. With the growing demand for WCD, the creation of a personalized energy distribution model based on a patient's anatomy, rather than a ‘one size fits all’ approach, is the need of the hour. Our proposed optimal electrotherapy assessment using biophysical modeling compares the efficiency of standard (Apex-Posterior) and non-standard (Front-Back) WCD electrode placement along with different plausible electrode locations variation throughout the torso, demonstrating significant differences in defibrillation efficacy associated with different strategies. The proposed approach of tuning defibrillation parameters coupled to a physical cardiac model that provides insights regarding the hemodynamic and electrophysiological changes at initiation or after the termination of an arrhythmic event could enable therapeutic device validation and testing, better patient stratification for ICD or similar invasive procedures, and creating subject-specific treatment plan providing a personalized approach toward cardiac care.

## Data Availability Statement

The original contributions presented in the study are included in the article/Supplementary Material, further inquiries can be directed to the corresponding author.

## Author Contributions

OM and AS conceptualized the overall study, methodology, and formulated defibrillation parameters. RB, AM, and AG contributed in data processing and deep learning architecture. OM and DR contributed in hemodynamic modeling. SK provided clinical viewpoints. OM, AS, RB, AM, DR, AG, and SK drafted the manuscript. All authors have contributed to the manuscript and approved the final version.

## Funding

This study received funding from Tata Consultancy Services Ltd. (TCS). The funder was not involved in the study design, collection, analysis, interpretation of data, the writing of this article, or the decision to submit it for publication.

## Conflict of Interest

OM, RB, DR, AM, AG, SK, and AS are employed by Tata Consultancy Service Ltd. (TCS).

## Publisher's Note

All claims expressed in this article are solely those of the authors and do not necessarily represent those of their affiliated organizations, or those of the publisher, the editors and the reviewers. Any product that may be evaluated in this article, or claim that may be made by its manufacturer, is not guaranteed or endorsed by the publisher.
