# Supplementary material for: Computational Model for Therapy Optimization of Wearable Cardioverter Defibrillator: Shockable Rhythm Detection and Optimal Electrotherapy
Source: Front Physiol. 2021 Dec 10;12:787180. doi: 10.3389/fphys.2021.787180 (PMC8703044; doi:10.3389/fphys.2021.787180)
Supplement: Supplementary file 1 [file Data_Sheet_1.PDF]

WCD working principle

A general WCD is composed of a garment, containing three self-gelling defibrillation patch electrodes, two on the back and one in the front, and 4 non-adhesive ECG electrodes (anterior/posterior/ right/left) connected to a monitoring unit and rechargeable lithium-ion batteries. The modelled WCD referred in this paper is the WCD model of Zoll electronics (WCD system, LifeVest, ZOLL, Pittsburgh, PA,USA). The sensing electrodes continuously monitors the patient's heart rhythm via two lead filtered ECG and can automatically deliver up to 5 posterior-anterior defibrillation shocks. Once an arrhythmia is detected, an alarm sequence starts. The device detection algorithm incorporates heart rate, template matching, and persistence of the event to classify shockable rhythm. The default ventricular tachycardia (VT) and ventricular fibrillation (VF) detection thresholds are set at 150 and 200 beats/min, respectively and the detection window is at-least 25 sec long. The device uses a bi-phasic shock waveform with programmable energy levels of up to 150 J (Reek et al., 2017).
